# A Case of Solitary Fibrous Tumor of the Greater Omentum Incidentally Detected during Preoperative Evaluation for Thyroid Cancer

**DOI:** 10.70352/scrj.cr.25-0193

**Published:** 2025-08-07

**Authors:** Rei Hatayama, Yasushi Rino, Yoshio Kure, Keisuke Komori, Yukiko Kanetsuna, Nobuyasu Suganuma, Roppei Yamada

**Affiliations:** 1 Department of Gastrointestinal Surgery, International University of Health and Welfare School of Medicine Atami Hospital, Atami, Shizuoka, Japan; 2 Department of Surgery, Yokohama City University, Yokohama, Kanagawa, Japan; 3 Department of Endocrine Surgery, Kure Clinic, Fujisawa, Kanagawa, Japan; 4 Department of Pathology, International University of Health and Welfare School of Medicine Atami Hospital, Atami, Shizuoka, Japan

**Keywords:** solitary fibrous tumor, case report, great omentum, thyroid cancer

## Abstract

**INTRODUCTION:**

Solitary fibrous tumor (SFT) is a mesenchymal tumor and accounts for less than 2% of all soft tissue tumors. It is most commonly found in the pleura and has a relatively good prognosis.

**CASE PRESENTATION:**

A 53-year-old woman was diagnosed with thyroid cancer and underwent a plain CT scan for preoperative examination. A mass measuring 23 mm in maximum diameter was incidentally found near the greater curvature of the stomach. Although its continuity with the stomach was unclear, an additional 18F-fluorodeoxyglucose positron emission tomography (FDG-PET)/CT scan revealed abnormal accumulation of Sstandardized uptake value (SUV) of max 4.2 at the same site. Percutaneous biopsy and endoscopic ultrasound-guided fine needle aspiration (EUS-FNA) were anatomically difficult, so a laparoscopic resection was performed as a diagnostic treatment. The specimen measured 28 mm in maximum diameter and was elastically hard. There was a proliferation of small- to medium-sized spindle cells, and no atypical cells were observed. No tumor components were found at the resection margin. The diagnosis of SFT was therefore made. The patient was discharged 2 days after surgery. Although it would have been difficult to diagnose the tumor based on preoperative imaging alone, we were able to perform minimally invasive tumor removal by laparoscopic surgery and make a diagnosis.

**CONCLUSIONS:**

Although the prognosis of SFT is generally good, its treatment has not been established. In particular, SFT originating from the greater omentum is extremely rare. We report this case along with some relevant literature.

## Abbreviations

EUS-FNA
: endoscopic ultrasound-guided fine needle aspiration
FDG-PET
: 18F-fluorodeoxyglucose positron emission tomography
GIST
: gastrointestinal stromal tumor
HPF
: high-power fields
SFT
: solitary fibrous tumor
SUV
: standardized uptake value

## INTRODUCTION

Differential diagnoses for intraperitoneal tumors include malignant lymphoma, gastrointestinal stromal tumor (GIST), and carcinoid, and we often encounter cases in which a definitive diagnosis cannot be made preoperatively. Solitary fibrous tumor (SFT) is a mesenchymal tumor that was first reported by Klemperer et al. in 1931,^1)^ and is reported to account for less than 2% of all soft tissue tumors.^2)^ If it could be resected, the prognosis is relatively good,^2)^ and in recent years, there have been occasional reports of laparoscopic resection.^3)^ It is most commonly found in the pleura,^2)^ but we report a case of SFT originating from the greater omentum, incidentally detected during the preoperative evaluation for thyroid cancer, along with some relevant literature.

## CASE PRESENTATION

A 53-year-old female patient was referred for further evaluation after an incidental finding during preoperative imaging for thyroid cancer. She had no significant complaints at presentation. Her past medical history included uterine fibroids and two prior cesarean sections. On physical examination, her height was 152 cm, weight 58.5 kg, and body mass index (BMI) was 25.32. The abdomen was flat and soft without tenderness, and a midline lower abdominal surgical scar was noted.

**Fig. 1:**
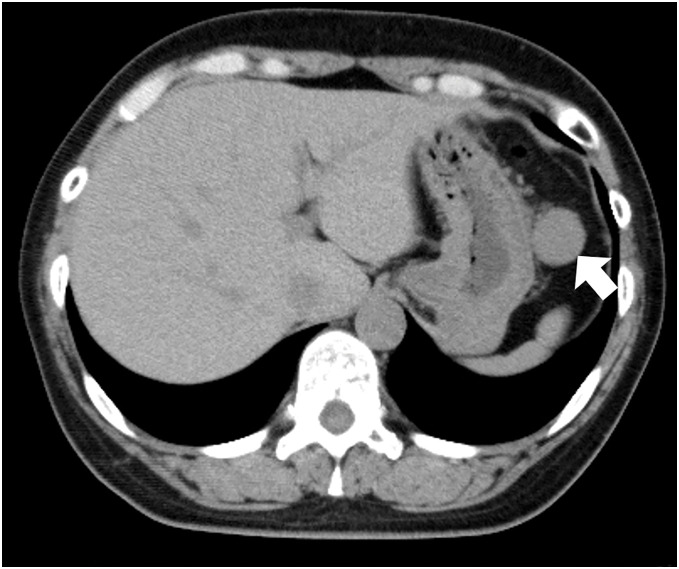
CT. Tumor near the upper part of the gastric body. The continuity with the stomach was not clear.

**Fig. 2:**
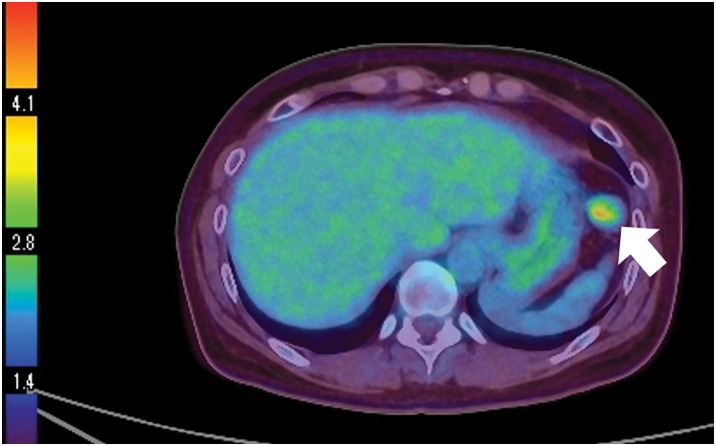
FDG-PET/CT. Abnormal accumulation of SUVmax 4.2 was observed in the mass observed on plain CT.

**Fig. 3:**
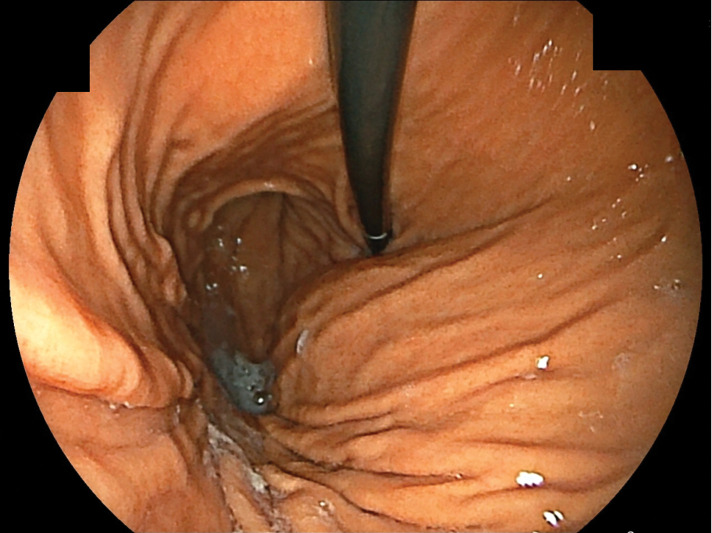
Upper gastrointestinal endoscopy. There were no findings suggestive of a submucosal tumor.

She was diagnosed with suspected thyroid cancer and a plain CT scan performed as a preoperative examination revealed a mass measuring 23 mm in maximum diameter near the greater curvature of the stomach (**Fig. 1**). Although the continuity with the stomach was unclear, FDG-PET/CT showed abnormal accumulation of SUVmax 4.2 at the same site, and the possibility of a malignant tumor could not be ruled out (**Fig. 2**). She was referred to our department for further examination and treatment. Blood tests showed no abnormal findings. Upper gastrointestinal endoscopy revealed no findings suggestive of a submucosal tumor (**Fig. 3**). Based on these findings, it would be difficult to diagnose using imaging tests alone, and resected it laparoscopically for pathologic diagnosis.

**Fig. 4:**
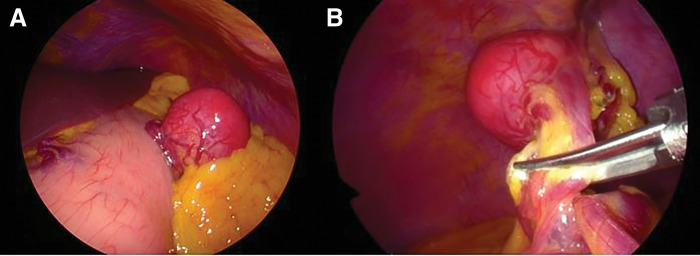
Surgical findings. (**A**) A well-mobile mass in the large reticulum near the upper part of the gastric body was observed. (**B**) The tumor was placed about 5 mm from the mass and resected with an ultrasonic coagulation incision device.

Under general anesthesia, a 12-mm port was inserted in the umbilicus and two 5-mm ports were inserted in the right upper quadrant, and endoscopic procedures were started using a total of three ports. A highly mobile mass was found in the greater omentum, into which branches of the left gastroepiploic artery and vein were flowing. No continuity with the stomach wall and other infiltration into the surrounding tissues were observed. And sufficient margins were secured through intraperitoneal procedures, after which the mass was resected using an ultrasonic coagulating and cutting device. The specimen was stored in a retrieval bag and extracted through the umbilical wound. The surgical time was 44 minutes with only a small amount of bleeding (**Fig. 4**).

**Fig. 5:**
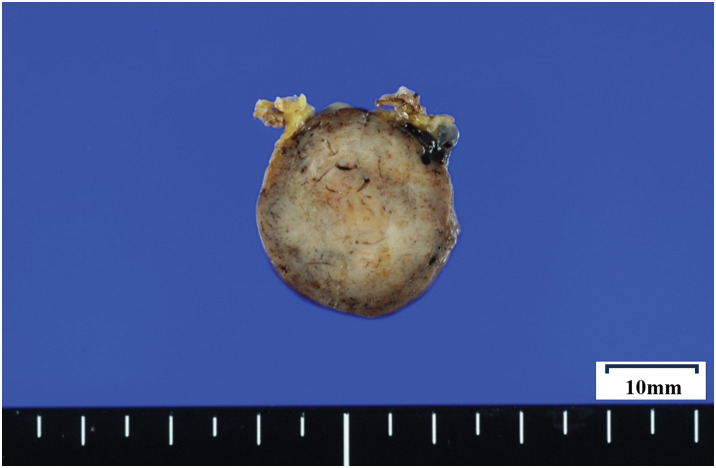
The resected specimen. It was 28 mm in maximum diameter.

**Fig. 6:**
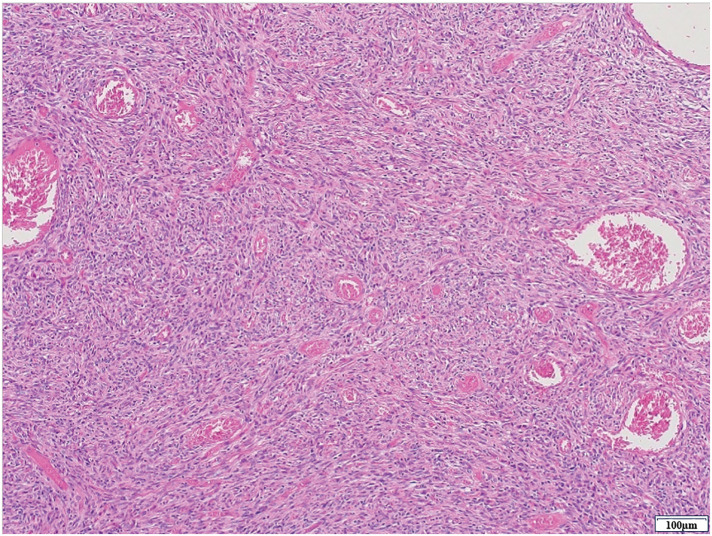
Microscopic findings of the tumor. Hematoxylin-eosin staining (×10).

The resected specimen measured 28 mm in maximal diameter and was elastically firm (**Fig. 5**). Histologically, moderate-sized spindle cells proliferated in a patternless pattern, with alternating hypercellular and hypocellular areas and scattered staghorn-like vessels. No nuclear atypia or mitotic figures were identified. The surgical margins were negative (**Fig. 6**).

**Fig. 7:**
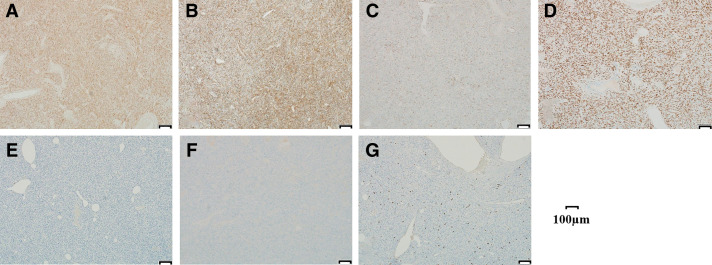
Immunostaining findings (×100). (**A**) Bcl-2, (**B**) CD34, (**C**) CD99, (**D**) STAT6, (**E**) c-kit, (**F**) DOG-1, and (**G**) MIB-1. Spindle cells were stained by Bcl-2, CD34, CD99, and STAT6.

Immunohistochemical staining showed positivity for CD34, STAT6, CD99, Bcl-2, and vimentin. Staining was negative for S100, desmin, SMA, c-kit, and DOG-1 (**Fig. 7**). The Ki-67 index was 4%, and no mitotic figures were observed in 50 high-power fields. Based on these findings, the tumor was diagnosed as a solitary fibrous tumor (SFT).

During the postoperative course, the patient was discharged 2 days after surgery without any complications. Approximately 1 month after surgery, a subtotal thyroidectomy and central node dissection was performed at the referring hospital. The pathology results of the resected specimen showed papillary thyroid carcinoma. Seven months after surgery, the patient had no recurrence of SFT.

## DISCUSSION

SFT is a mesenchymal cell-derived tumor that was first reported by Klemperer et al. in 1931 as a pleural lesion tumor.^1)^ It is a relatively rare disease with an incidence rate of 2.8 per 100000 people. The most common age for SFT is in the 60s and 70s, with no gender predilection.^4)^ In the WHO classification, SFT is classified as an intermediate-grade tumor with a low rate of metastasis among fibroblastic/myofibroblastic tumors. The most common site of occurrence is the chest, where it is said to account for 80% of cases.^2)^ In recent years, there have been occasional reports of SFT originating from the greater omentum outside the pleura.

SFT might show low accumulation in benign cases and high accumulation in malignant cases on FDG-PET/CT.^5)^ Li et al. reported that the threshold SUVmax for predicting malignant GIST is 2.6–3.0.^6)^ Carcinoids do not have as high a tumor proliferation activity as carcinomas, so although the detection rate with FDG-PET/CT is 25%–73%, it is said that there is a possibility of detection in highly malignant carcinoids.^7)^

However, it is difficult to exclude other diseases and diagnose SFT by imaging tests alone, and a definitive diagnosis is made by histopathological tests including immunohistochemical tests. The histopathological images are mainly composed of spindle or oval cells and collagenous stroma, and show a patternless pattern with different cell densities and arrangement patterns depending on the location.^8)^ Immunohistochemical staining shows high positivity for CD34, CD99, STAT6, Bcl-2, and Vimentin.^7^,^9–11)^ Some SFTs are malignant and cause recurrence or distant metastasis. The incidence of malignant SFT is 10%–15% in pleural primary tumors and 2% in extrapleural primary tumors.^1,12)^ Risk factors include^1)^ tumor diameter exceeding 10 cm,^2)^ mitotic figures of 4–10 high-power fields (HPF) or more,^3)^ high cell density,^4)^ and hemorrhage or necrosis.^8)^ Although neither of these conditions applied to this case, there have been reported cases of recurrence despite no evidence of malignant risk,^12)^ as well as cases of recurrence more than 10 years after surgery.^13)^ Although the present case originated from the greater omentum, similar recurrences have been reported even in more common pleural SFTs that were histologically diagnosed as benign after surgical resection.^14)^ Given that SFT is a rare tumor with no established treatment guidelines, long-term follow-up is considered essential. Surgical resection is the fundamental treatment for SFT, and there is no established chemotherapy and radiation therapy.^2)^

As mentioned above, SFT arising from the greater omentum is very rare. A search for the keywords “solitary fibrous tumor” and “omentum” in NPO Japan Medical Abstracts Society revealed 12 cases of SFT originating from the greater omentum, and 13 cases, including this case, were evaluated (**Table 1**).^3,15–25)^

**Table 1:** Case reports of SFT originating from the greater omentum

Author	Year	Age/Gender	Size (cm)	Operation	Immunostaining positive	Malignant risks	Prognosis (month)
Watanabe^15)^	1998	66/M	11	Open	CD34	(+) (Size >10 cm)	NA
Miyazaki^16)^	2002	69/M	9.5	Open	CD34, Bcl-2, S-100	NA	Alive (17)
Nakata^17)^	2012	71/M	3.5	Open	CD34	NA	Alive (NA)
Senda^18)^	2014	45/F	7.5	Open	CD34, Bcl-2	NA	Alive (9)
Osawa^19)^	2014	32/F	4.8	Laparoscopy	CD34, Bcl-2	NA	Alive (12)
Ishi^20)^	2014	41/M	3	Laparoscopy	CD34	NA	Alive (5)
Michiura^21)^	2016	36/M	9	Open	CD34, Bcl-2	NA	Alive (8)
Ozaki^22)^	2017	32/F	10	Laparoscopy	CD34, STAT6	NA	Alive (7)
Suzuki^23)^	2019	45/F	5.5	Open	CD34, Bcl-2	NA	Alive (3)
Yamane^24)^	2020	69/F	6.5	Laparoscopy	CD34, STAT6	NA	Alive (6)
Eltawil^25)^	2021	63/M	12	Open	CD34, Bcl-2	(+) (Size >10 cm)	Alive (6)
Marunaka^3)^	2024	47/F	2.8	Laparoscopy	CD34, STAT6	NA	Alive (6)
Our case		53/F	2.8	Laparoscopy	CD34, Bcl-2, STAT6, CD99	NA	Alive (7)

NA, not available

The male-to-female ratio was 6:7. All cases were completely resected, and 2 cases were at risk of malignancy. Regarding the surgical procedure, open surgery was performed in 7 cases and laparoscopic surgery in 6 cases. The average tumor diameter was 8.2 and 4.9 cm in open and laparoscopic surgery, respectively. The largest tumor resected laparoscopically was 10 cm and was retrieved under laparoscopy by being cut into small pieces in a retrieval bag.^22)^ In the present case, the tumor was relatively small at 2.8 cm in diameter, making it one of the smaller lesions reported in the literature. As a result, a minimally invasive laparoscopic resection could be performed. Regarding immunohistochemical analysis, although several markers have a high positivity rate, there are no universally established criteria that define a mandatory diagnostic marker. STAT6, now considered a key marker for SFT, was first recognized as being associated with SFT in 2013. Therefore, earlier case reports often lack STAT6 testing. The selection of immunohistochemical markers appears to vary depending on the time period and the preferences or practices of individual pathologists. In addition, when preoperative imaging diagnosis is difficult, it may be useful to observe the lesion under laparoscopy and then select the surgical procedure based on the findings.

Of note, synchronous occurrence of papillary thyroid carcinoma was also observed. Although the literature search was challenging, a case of intra-abdominal SFT concurrently associated with papillary thyroid carcinoma was reported by Boleko et al.^26)^ In that case, the SFT exhibited extensive intra-abdominal involvement with infiltration into multiple organs, and the primary site was unknown. Complete resection was not achieved, and the pathological findings supported the diagnosis of malignant SFT. While no direct association with thyroid cancer was demonstrated, the authors speculated that a genetic background might be involved. Meanwhile, Ben Thayer et al. and Papi et al. have reported a case of concurrent papillary thyroid carcinoma and primary SFT of the thyroid, and concluded that this coexistence is likely coincidental.^27,28)^ Similarly, in our case, no clear relationship between the thyroid cancer and the omental SFT was identified based on pathological findings. In addition, the 2024 Guidelines for the Management of Thyroid Tumors by the Japan Association of Endocrine Surgeons do not mention any association or coexistence between papillary thyroid carcinoma and other types of tumors, including solitary fibrous tumors.^29)^ Further accumulation of similar case reports is necessary to clarify potential associations.

## CONCLUSIONS

Primary omental SFTs, incidentally detected during the preoperative evaluation for thyroid cancer, are very rare. Further accumulation of cases and longer follow-up are needed to clarify the appropriate treatment and postoperative surveillance methods.

## DECLARATIONS

### Funding

None.

### Authors’ contributions

RH, YR, KK, and RY contributed to surgery.

YK contributed to first consultation.

YK contributed to pathological diagnosis.

NS contributed to endocrine surgical consultation.

The final manuscript was read and approved by all the authors.

All authors agree to be responsible for all aspects of the study.

### Availability of data and materials

Not applicable.

### Ethics approval and consent to participate

Not applicable.

### Consent for publication

Consent has been obtained from the patient for publication of this study.

### Competing interests

None.
